# Antecedents of Nurse Managers’ Work Engagement: A Cross-Sectional Study

**DOI:** 10.3390/healthcare11091336

**Published:** 2023-05-06

**Authors:** Andrea Forster, Clemens Koob

**Affiliations:** Department of Health and Nursing, Catholic University of Applied Sciences Munich, Preysingstraße 95, 81667 Munich, Germany

**Keywords:** nurse managers, work engagement, demands, resources, job demands–resources theory

## Abstract

The responsibilities of nurse managers are complex. Their actions are crucial to providing the best possible care to patients and to the success of health care organizations. Thus, nurse managers’ work engagement is essential. However, understanding of the antecedents of nurse managers’ work engagement is lacking. The job demands–resources theory posits that work engagement is contingent upon job resources and demands. Therefore, the aim of this study was to explore which job demands and resources exert a major influence on nurse managers’ work engagement. Considering the literature, job resources and demands potentially relevant to nurse managers’ work engagement were identified. To investigate the associations between these potential antecedents and nurse managers’ work engagement, the study employed a cross-sectional survey. The dataset for analyses comprised 408 nurse managers in Germany and was analyzed by multiple linear regression. The study variables accounted for 26% of the variance in nurse managers’ work engagement. Positive associations were detected between the job resource of empowering leadership and nurse managers’ work engagement. Regarding job demands, lack of formal rewards and work–life interferences had negative effects on work engagement. The findings suggest that the job demands–resources theory can explain nurse managers’ work engagement. However, not all job resources and demands considered were determined to be influential. In conclusion, empowering leadership should be promoted in the work environment of nurse managers. Nurse managers should be provided engaging financial and nonfinancial rewards. Work–life interferences should be systematically mitigated.

## 1. Introduction

Nurse managers have multiple responsibilities, such as managing resources, aligning the goals of their department with the strategic goals of the healthcare organization, and ensuring that patients receive the best possible care. As an integral part of their responsibilities, they have to collaborate closely with different professional groups within a healthcare organization and hold an important interface function [1]. One of their core responsibilities is to manage caregivers effectively. In this way, nurse managers can help ensure that patients receive the care they need [2]. Effective management of caregivers includes positively shaping caregivers’ work environment to foster their job satisfaction, productivity and performance, as well as health [3]. The COVID-19 pandemic added to the complexity of nurse managers’ responsibilities and tasks, e.g., by further exacerbating staff shortages, additional workload and the need to support caregivers in distress [4,5].

Given these complex responsibilities and tasks, nurse managers’ work engagement is essential. Work engagement denotes a positive, fulfilling, work-related psychological state characterized by vigor, dedication, and absorption [6]. Previous research has regularly determined work engagement to be associated with better health and, in particular, higher job performance, positive work behaviors that go beyond contractual obligations (Organizational Citizenship Behaviors), and fewer counterproductive work practices [7,8].

From a health care management perspective, therefore, the question arises as to the critical antecedents of nurse managers’ work engagement. With respect to caregivers, there are a number of studies on work engagement including its antecedents [9]. More recently, factors influencing work engagement during the pandemic have also been studied more specifically for this group of employees [10,11,12].

In marked contrast, and despite the tradition of academic work in nursing, there are only few publications related to the work engagement of nurse managers. Existing studies concerning nurse managers focus primarily on other work-related attitudes such as job satisfaction [13] or health-related aspects such as job stress [14,15], burnout and well-being [16]. The few studies addressing work engagement are either limited to Belgium or based on a small sample and present partially different results. Van Bogaert et al. [17] conducted a cross-sectional study that included 365 nurse managers from 17 Belgian acute care hospitals. The study explored the influence of role-related, work-related, and organizational factors on various outcome variables, including work engagement. It was determined that role conflict, role ambiguity, and perceived lack of role importance along with high demands, low work control, and social support as predictors explained 34% of the variance in nurse managers’ work engagement. Adriaenssens et al. [18] investigated associations between various factors such as work control, social support, work demands, collaboration with medical staff, and personal characteristics and work engagement. The quantitative study, also conducted in Belgium in 11 hospitals with 318 nurse managers, reported control over one’s own work and social support to be positively related to work engagement, while the other factors did not reveal statistically significant effects. Conley [19] used a sample of 47 nurse managers to investigate strategies used to achieve and sustain engagement in acute care settings. Expert communication, autonomy, and influence were identified as drivers of engagement. Given this limited body of research, it can be stated that the understanding of the antecedents of nurse managers’ work engagement is lacking. We address this knowledge gap in this paper.

## 2. Theoretical Framework

Investigating antecedents of nurse managers’ work engagement requires an appropriate theoretical framework. To examine work engagement, the job demands–resources theory is commonly drawn on [8]. This theory assumes that every job involves both demands and resources. Excessive job demands are expected to reduce work engagement. In contrast, job resources, such as physical, psychological, social, and organizational aspects of work that are functional for achieving work-related goals, are supposed to have a positive effect on engagement [8]. According to the job demands–resources theory, the question of the antecedents of nurse managers’ work engagement thus points to the question of which job demands and job resources exert a major influence on work engagement.

### 2.1. Nursing Profession-Related Job Resources and Demands

A starting point to determine potential candidates for such factors is to first identify job demands and resources that have been generally determined to be important to members of the nursing profession. In an integrative review of reviews, Broetje et al. [20] identified six key resources and three key demands specific to nursing professionals. Among the key resources, first is the *autonomy* to make work-related decisions and to exert control over one’s work. By *interpersonal relationships*, supportive relationships are addressed, especially between colleagues of the same or similar hierarchical levels. *Professional resources* represent the tangible (e.g., equipment, financial resources) and intangible (e.g., access to information, task organization) resources that support good work. The other three resources relate to aspects of leadership that, as Broetje et al. [20] suggest, might not be entirely distinct: *supervisor support* addresses social support by direct leaders (e.g., constructive feedback), *fair and authentic management* refers to leaders’ fairness and trust, and *transformational leadership* emphasizes leadership behaviors that are inspirational (e.g., articulation of a meaningful vision), individualized (e.g., consideration of an individual subordinate’s current needs), exemplary (e.g., acting as an ethical role model) and intellectually stimulating (e.g., providing subordinates with challenging new ideas). Among key demands, *work overload* refers to work and time pressure and inadequate staffing, while *lack of formal rewards* concerns remuneration that is perceived as unfair and insufficient opportunities for development. Finally, *work–life interferences* include imbalances between work and personal or family life (e.g., change in private plans due to work obligations), but also refer to unfavorable duty or assignment scheduling.

### 2.2. Nursing Management-Specific Job Resources and Demands

As the perceptions of job-related parameters can differ between staff nurses and nurse managers [21], it seems mandatory to additionally elicit potential nursing management-specific job resources and demands. Information in this regard can be obtained on the one hand from the studies on nurse managers’ work engagement already mentioned in the introduction [17,18,19]. On the other hand, information can be derived from studies and reviews that have investigated determinants of job satisfaction or job stress among nurse managers [13,14,15]. A synthesis of these studies’ findings first corroborates that autonomy, interpersonal relationships among colleagues, professional resources, and supervisor support could potentially be job resources relevant to nurse managers’ work engagement. Beyond that, these studies point to four other candidates for potentially important job resources: *transparent information and communication* [19], *effective relationships with nursing staff* [13], *collegial relationships with medical staff* [13], and *empowering leadership* [13]. The resource category of empowering leadership here relates to leadership behaviors that enable nurse managers to attain optimal outcomes for staff, patients, and the organization [22,23] and has conceptual similarities with the authentic as well as transformational leadership mentioned above by Broetje et al. [20,24]. Regarding job demands, the studies underpin the potential relevance of the job demands already identified by Broetje et al. [20]. In addition, they also point to *role conflicts* as a further possible management-specific job demands category [14,15].

### 2.3. Job Resources and Demands following from the COVID-19 Pandemic

Since the work environment of nurse managers has recently been under the influence of the COVID-19 pandemic, a third theoretical lens is to look for possible job resources and job demands that may have emerged because of the pandemic situation and may also be relevant in the post-pandemic future. Quantitative [25] and qualitative studies [26,27,28] that have examined nurse managers’ experiences during the pandemic underscore, first, the potential relevance of the aforementioned job resources of interpersonal relationships, supervisor support, and transparent information and communication. Second, these studies again stress the possible relevance of the already identified job demands in terms of work overload, work–life interferences, and role conflicts. Finally, these studies point to another potentially important job demand category of nurse managers in the form of *emotional demands* that arise, for example, from their own worries and fears, but also from the fact that managers must support staff nurses in coping with anxiety and concerns.

**Figure 1:**
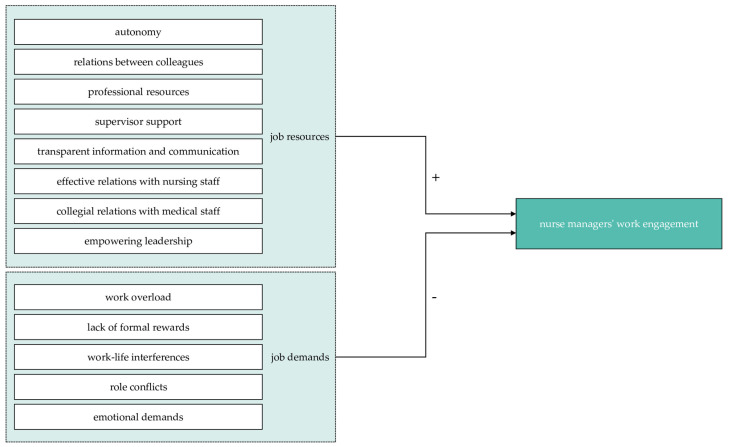
Investigated relationships.

In summary, (1) autonomy, (2) relations between colleagues, (3) professional resources, (4) supervisor support, (5) transparent information and communication, (6) effective relations with nursing staff, (7) collegial relations with medical staff, and (8) empowering leadership can be derived from the literature as job resources potentially exerting positive influence on nurse managers’ work engagement. Job demand categories that potentially reduce engagement emerge from the literature as (1) work overload, (2) lack of formal rewards, (3) work–life interferences, (4) role conflicts, and (5) emotional demands. Figure 1 shows the investigated relationships.

## 3. Materials and Methods

### 3.1. Study Design

To investigate the associations between the identified job demands and resources and nurse managers’ work engagement, an analytical cross-sectional study design was adopted. A structured questionnaire with measures that have proven valid and reliable in prior studies was used for data collection. All questions were asked in the German language. The SoSci Survey software was used to implement the questionnaire online. Before the survey was fielded, a pre-test was performed, and resulting comments were included.

### 3.2. Participants

Nurse managers in middle or lower management positions who worked in hospitals or geriatric care facilities in Germany were eligible to participate in the study. Eligible participants included ward, residential area and department managers, nursing area and nursing service managers, managers in comparable positions, as well as their respective deputies. Individuals in managerial positions in outpatient care services were not included, as were healthcare organizations’ top managers.

The study is based on a convenience sample. To recruit participants, the survey link was first shared via social media (i.e., nursing management-related WhatsApp and Facebook groups). Second, the link was sent to 230 facility managers of geriatric care facilities and to nursing directors of 190 hospitals with the request to forward it to members of the target group. The geriatric care facilities were selected at random from the directory of the German Seniors Portal and the hospitals from the directory of the German Hospital Federation (DKG). To obtain an adequate sample size in the comparatively difficult-to-reach target group, the link was also sent to the members of the German Federal Association of Nursing Management. This association can be considered to be one of the main national professional organizations in the nursing management domain and has approximately 1000 members working as managers in the nursing and health care sector. The addressees were asked to participate themselves if they were eligible for participation or to forward the link to appropriate contacts. To preserve the anonymity of participants, it was not tracked which of the three recruitment routes the study participants originated from.

### 3.3. Measures

#### 3.3.1. Work Engagement

For measuring nurse managers’ work engagement as outcome variable, the ultra-short version of the Utrecht Work Engagement Scale with three items (UWES-3) was used which has shown good reliability and validity [29]. Items were measured on a 7-point Likert scale ranging from “never” to “always”. An example item was “I am enthusiastic about my job”.

#### 3.3.2. Job Resources

For measuring the extent to which nurse managers can choose how they carry out their work (*autonomy)*, the autonomy and control scale from Haynes et al. [30] was used. The six-question scale was translated into German for this study according to Beaton et al.’s [31] recommendations. An example question was “To what extent do you determine the methods and procedures you use in your work?”. Response options on a 5-point Likert scale ranged from “not at all” to “a great deal”.

*Professional resources, supervisor support, effective relationships with nursing staff, collegial relationships with medical staff*, and *empowering leadership* were operationalized using the corresponding domains of the Nurse Manager Practice Environment Scale (NMPES) that was determined to have good psychometric qualities [23]. Translation of the items into German followed the recommendations of Beaton et al. [31]. The wording of some items was slightly adjusted to ensure an optimal fit for the different groups of participants included in this study. Nurse managers’ professional resources were captured with four items, an example was “The budget allocations for my patient care area(s) are adequate”. Supervisor support was assessed with six items, e.g., “I receive feedback from my immediate supervisor that helps me develop my leadership skills”. Effective relationships with nursing staff were measured with three items, including, e.g., “My unit staff works with me to resolve patient care issues”. Collegial relationships with medical staff were captured with three items, an example item is “Physicians understand my role as nurse manager”. Empowering leadership was operationalized with the 15 corresponding NMPES items, including, e.g., “I am empowered to do my job” and “My executive nurse administrators encourage innovative solutions to problems”. All NMPES items were measured on 6-point Likert scales ranging from “strongly disagree” to “strongly agree”.

Finally, *interpersonal relations between colleagues* and *transparent information and communication* were measured using the German version of the Copenhagen Psychosocial Questionnaire (COPSOQ), which showed adequate psychometric properties [32]. *Interpersonal relations between colleagues* were operationalized with the two-question sense of community subscale and the two questions of the social support subscale that refer to colleagues. An example is “Is there a good atmosphere between you and your colleagues?”. Participants were instructed to answer these questions in relation to colleagues at a comparable hierarchical level. *Transparent information and communication* were captured with the corresponding two-items subscale, an example item was “At your place of work, are you informed well in advance concerning, for example, important decisions, changes, or plans for the future?” All COPSOQ items were measured on 5-point Likert scales.

#### 3.3.3. Job Demands

To capture any *lack of formal rewards*, the seven-item reward subscale of the German short version of the Effort-Reward Imbalance Questionnaire (ERI-S) was used [33]. An example item was “Considering all my efforts and achievements, my salary/income is adequate”. Items were coded in a way that higher numerical values corresponded to a stronger lack of formal rewards. *Work overload* was measured with the three-item effort subscale of the ERI-S. An example item was “I have constant time pressure due to a heavy workload”. All ERI-S items were measured on a 4-point agreement scale. Prior research showed adequate psychometric properties of the ERI-S [33].

The remaining job demands were operationalized using the COPSOQ. *Work–life interferences* were assessed using the four-item work–privacy conflict subscale. An example item was “The demands of my work interfere with my home and family life.” *Emotional demands* were assessed with the two items of the corresponding subscale, an example was “Is your work emotionally demanding?”. *Role conflicts* were measured with the three corresponding COPSOQ items, an example was "Are contradictory demands placed on you at work?”. All COPSOQ items were measured on 5-point Likert scales ranging from “to a very small extent” to “to a very large extent” and from “never, hardly ever” to “always”.

#### 3.3.4. Baseline Data and Control Variables

To describe the sample and as possible confounders, participants’ age, gender, management position, and type of healthcare organization were recorded. In addition, the qualification level for the managerial position, whether the respondents also worked in direct nursing care, the span of supervision (number of subordinate employees), for how many years in total the respondents had been employed in the nursing field, and how many years of these they worked in a managerial position were included as controls.

### 3.4. Bias and Data Quality

To address common method bias concerns, we followed Podsakoff et al.’s [34] recommendations for procedural remedies. The questionnaire was sectioned, with instructions provided between sections, to psychologically separate the measures. Response options were verbalized to ensure consistency of understanding. To avoid method bias from common scale properties, different response scales and scale point labels were used, always in accordance with the original survey instruments. The items were kept specific to mitigate ambiguity. Anonymity was ensured to reduce social desirability bias, and the questionnaire was kept as brief as possible to encourage accurate answers.

To alleviate data quality concerns, potential study participants were asked screening questions to verify inclusion criteria. To ensure high-quality responses, we excluded speeding participants, i.e., participants who responded extremely fast, since careless responses are identified most reliably by questionnaire completion time [35]. We applied the DEG_TIME indicator excluding respondents with more than 100 malus points for fast completion [36], the relative speed index excluding respondents with values of 2.0 or higher [35], and excluded cases that needed less than half the median processing time to complete the questionnaire [37].

### 3.5. Study Size

The required sample size was determined by an a priori power analysis using G*Power (version 3.1). As is common in multiple linear regression analyses, Cohen’s f^2^ was used as a measure of the joint effect of all variables [38]. f^2^ equals the proportion of variance explained by the predictors divided by the residual variance, i.e., f^2^ = R^2^/(1-R^2^). According to Cohen [38], f^2^ = 0.02 denotes a small effect, f^2^ = 0.15 denotes a moderate effect, and f^2^ = 0.35 signifies a large effect. According to previous studies in the nursing sector on the relationships between job resources and demands and work engagement (e.g., [9]), a medium to large effect was conceivable. For this reason, an expected effect size of at least f^2^ = 0.15 was set for the power analysis. With an error probability of α = 0.05, an effect size of f^2^ = 0.15, and a target power of 1-β = 0.80, the a priori power analysis for the multiple linear regression with 29 predictors planned in the main analysis indicated a required total sample size of 184 participants.

### 3.6. Statistical Analyses

SPSS Statistics 28 was used for all analyses. First, the data set was cleaned by excluding all cases that did not meet the inclusion criteria or could be classified as a speeder. Next, an adequate method for treating missing data was determined by analyzing the underlying mechanism. Little’s test indicated (*p* < 0.001) that the data were not missing completely at random (MCAR). However, there were only missing values in predictors because we used mandatory answering in the outcome variable. Under these conditions, listwise deletion of cases is practicable, even if the MCAR condition is not met. Therefore, and to avoid problems of imputation procedures, listwise deletion was chosen [39]. Subsequently, the sample was described, and means, standard deviations, correlations and Cronbach’s alphas of the study variables were calculated.

We used multiple linear regression analysis to examine the relevance of the identified job resources and demands for nurse managers’ work engagement. The assumptions of regression analysis were tested before the main analysis. We assessed linearity between the outcome and the predictor variables using partial residual plots of predictor variables. The plots exhibited only minor deviations from linear relations. The highest value of the variance inflation factor, 3.51, was below the recommended threshold of 10 [40], so there was no indication for collinearity concerns. Inspection of histograms and P-P plots showed no evidence of violating the normality assumption of residuals. A Shapiro–Wilk test (*p* = 0.52) also did not indicate a violation of the normality assumption of residuals. The Breusch–Pagan test (χ^2^(1) = 6.22, *p* < 0.05) indicated heteroscedasticity. Therefore, we proceeded with the analysis of the regression model and followed the recommendation of Hayes and Cai [41] to employ HC3 as robust standard error estimator in the regression to account for heteroscedasticity. All study variables were entered into the regression equation on the same step. Categorical control variables were recoded into dummy variables for regression to allow them to be considered as independent variables. In each case, j-1 dummy variables were included for a variable with j categories.

Cohen’s f^2^ was used to classify the joint effect of job resources and demands on work engagement, with 0.02 denoting a small effect, 0.15 denoting a moderate effect, and 0.35 signifying a large effect. Standardized regression coefficients were used as effect size index to classify the effects of each individual job resource and demand. Absolute values less than 0.2 indicate small effects, values between 0.2 and 0.5 indicate moderate effects, and values greater than 0.5 indicate strong effects. A *p*-value < 0.05 was considered significant.

## 4. Results

### 4.1. Participant Data

A total of 605 responses were collected. The final sample comprised N = 408 nurse managers after eliminating responses that failed to fit with the inclusion criteria or did not meet the quality checks. Table 1 displays the characteristics of the sample.

No data were available on the structure of the population of nurse managers in German hospitals and geriatric care facilities, so the sample could not be compared to the population. However, the characteristics of the sample corresponded well with the objectives of the study. The study participants were nurse managers from diverse backgrounds.

### 4.2. Descriptive Statistics

According to related guidelines [42,43], Table 2 lists the means, standard deviations, the full correlation matrix, and the Cronbach’s alphas of all study variables. The Cronbach’s alphas of the multi-item scales ranged from 0.71 to 0.94, surpassing the acceptable level of 0.70 [40].

Consonant with job demands–resources theory, statistically significant positive correlations were detected between all job resources and work engagement (see Table 2), with correlation coefficients (r = 0.174 to 0.425, *p* < 0.01) indicating weak to moderate relationships between the variables [38]. In line with the theory, the job demands were determined to correlate significantly and negatively with nurse managers’ work engagement, with the correlation coefficients (r = −0.133 to −0.409, *p* < 0.01) indicating weak to moderate associations [38]. The only exception were emotional demands, which were not determined to correlate significantly with work engagement (r = 0.010, *p* = 0.836).

### 4.3. Results of the Main Analysis

The results of the multiple linear regression with nurse managers’ work engagement as the dependent variable are shown in Table 3. The study variables explained a substantive proportion of the variance in nurse managers’ work engagement (adjusted R^2^ = 0.260, *p* < 0.001). According to Cohen’s [38] conventions, this corresponds to a large joint effect of the studied predictors on work engagement (f^2^ = 0.351).

Based on the job demands–resources theory, positive relationships were expected between the job resources derived from the literature and the work engagement of nurse managers. The regression coefficient showed that empowering leadership was significantly and positively associated with work engagement, as hypothesized (β = 0.276, t = 3.176, *p* < 0.01). With a value in the range between 0.2 and 0.5, the standardized regression coefficient indicated a moderate effect. However, contrary to expectations, the results did not suggest significant relationships between the other investigated job resources and nurse managers’ work engagement. No significant associations with nurse managers’ work engagement were detected for autonomy (β = 0.087, t = 1.678, *p* = 0.094), interpersonal relationships between colleagues (β = 0.073, t = 1.370, *p* = 0.171), professional resources (β = −0.045, t = −0.688, *p* = 0.492), supervisor support (β = 0.040, t = 0.511, *p* = 0.610), transparent information and communication (β = −0.055, t = −0.755, *p* = 0.450), effective relationships with nursing staff (β = 0.075, t = 1.313, *p* = 0.190), and collegial relationships with medical staff (β = 0.032, t = 0.635, *p* = 0.526), respectively. Overall, the presumed relationships between nurse managers’ job resources and work engagement are thus only partially supported by the study data.

Regarding job demands, negative associations with work engagement were expected from a theoretical perspective. This expected relationship was confirmed for a lack of formal rewards. A greater lack of formal rewards was associated with significantly lower work engagement (β = −0.151, t = −2.406, *p* < 0.05). With an absolute value smaller than 0.2, the standardized regression coefficient indicated a small effect. Work–life interference also emerged as a significant predictor of work engagement. Greater perceived work–life interferences were associated with significantly lower work engagement (β = −0.120, t = −2.137, *p* < 0.05). With an absolute value of less than 0.2, the standardized regression coefficient also indicated a small effect here. In contrast, and other than expected, work overload (β = 0.007, t = 0.114, *p* = 0.909), role conflicts (β = −0.041, t = −0.692, *p* = 0.489), and emotional demands (β = 0.077, t = 1.477, *p* = 0.141) were not determined to be significant predictors of nurse managers’ work engagement. Overall, the suggested relationships between job demands and the work engagement of nurse managers find partial support from the study data.

## 5. Discussion

### 5.1. Theoretical Implications

Few studies have examined what determines nurse managers’ work engagement. These studies were either based on small samples, treated work engagement only peripherally, did not have a concise theoretical basis, or focused on Belgium. Furthermore, all these studies were conducted before the COVID-19 pandemic, so that constellations that may have changed due to the pandemic are not covered by previous studies. Therefore, we used the job demands–resources approach as theoretical framework and quantitatively and systematically examined the impact of nurse managers’ job demands and job resources on their work engagement.

Our findings first add to the body of the literature on the work environment of nurse managers. We used three perspectives, a general profession perspective, a nursing management-specific perspective, and a pandemic perspective, to synthesize potential key categories of nurse managers’ job resources and job demands. Our thematic analysis identified (1) autonomy, (2) interpersonal relations between colleagues, (3) professional resources, (4) supervisor support, (5) transparent information and communication, (6) effective relationships with nursing staff, (7) collegial relationships with medical staff, and (8) empowering leadership as potential major job resources. (1) Work overload, (2) lack of formal rewards, (3) work–life interferences, (4) role conflicts, and (5) emotional demands emerged as potential major job demands.

Second, the results of the present study corroborate the finding that the job demands–resources theory, which has been examined in other worker populations including staff nurses, is also capable of explaining nurse managers’ work engagement. Overall, the job resources and job demands studied had a considerable impact on nurse managers’ work engagement. They jointly were able to explain 26% of the variance in work engagement. Our model is somewhat less explanatory than the analysis by Bartsch et al. [10], who were able to explain 36% of the variance in staff nurses’ work engagement. However, our study revealed a strong effect (f^2^ = 0.35). Future investigations could include further factors that might influence the work engagement of nurse managers, such as nurse managers’ personal resources, to account for an even higher share in variance.

Furthermore, it became apparent that, regarding work engagement as an outcome, not all the job resources and demands distilled from the literature seem to have an impact. In terms of job resources, empowering leadership was determined to affect the work engagement of nurse managers significantly and positively. The effect could be classified as moderate with reference to the standardized regression coefficient. This result extends previous research, which has reported associations between empowerment and other outcomes such as nurse managers’ intention to stay (positive) [44], role satisfaction (positive) [45], burnout (negative), physical health (positive) and mental health (positive) [46]. From our result, it is also clear that empowerment is important not only to staff nurses’ [47] but also nurse managers’ work engagement. Beyond the influence of empowering leadership, we detected no effects of the examined work-related resources (professional resources, autonomy), social resources (supervisor support, relations between colleagues, with nursing staff, or with medical staff), and informational resources (transparent information and communication) on nurse managers’ work engagement. This is noteworthy because others have demonstrated positive effects of some of these factors on the work engagement of staff nurses [10,12] and also nurse managers [18]. Our finding is also notable since empowering leadership is only one form of structural empowerment [22]. Other forms of structural empowerment include access to information, to support, and to resources [48], aspects that were also examined as resource categories in this study but do not appear to be of corresponding importance to nurse managers’ work engagement. Following up on this, further research is warranted. It is conceivable, for example, that specific combinations of aspects of structural empowerment could unfold impact on nurse managers’ work engagement so that investigating potential interaction effects between the corresponding job resources could enhance theory at this point.

Regarding job demands, lack of formal rewards and work–life interferences emerged as statistically significant for nurse managers’ work engagement. Considering the standardized regression coefficients, both factors unfolded small negative effects, while empowering leadership had a moderate positive effect. This result ties with the assumption of the job demands–resources theory that work engagement is stronger related with job resources than with job demands [29]. Previous research has already shown that lacking formal rewards negatively affect staff nurses’ work engagement [10]. Our finding goes beyond these reports by highlighting that this is also the case for the work engagement of nurse managers. Our result that work–life interferences have a detrimental effect on nurse managers’ work engagement fits well with previous research in which such interferences were determined to be associated with nurse managers’ organizational turnover intentions [49]. Further, our study detected no negative effects of work overload, role conflicts, and emotional demands on engagement. Rather, these job demands did not show any effect at all on nurse managers’ work engagement. While this finding is generally in accordance with the job demands–resources theory, according to which hindering demands can either be negatively related or unrelated to work engagement [29], it is nevertheless notable, as other studies determined these job demands to negatively affect aspects such as nurse managers’ stress or turnover intentions [14,44]. Therefore, further investigations are warranted. For example, it might be beneficial to examine whether the nurse managers’ “professional sense of duty” [50] or “leadership sense of duty” [51] moderate the impact of the aforementioned job demands on work engagement.

### 5.2. Practice Implications

This study has important implications for health care management practice. The strength of the association between the investigated job resources and job demands and nurse managers’ work engagement varied, as the standardized regression coefficients indicated. This suggests that leaders of healthcare organizations may prioritize improvements in job resources and job demands in the order of importance to nurse managers’ work engagement proposed in the present study.

Following this, the first recommendation is to promote empowering leadership and related positive leadership behaviors in the work environment of middle- and lower-level nurse managers, as this has emerged as the strongest influencing factor on their work engagement. Measures could include implementing appropriate practices for hiring and promoting senior managers and direct supervisors of nurse managers. For example, personal factors that might promote empowering leadership behaviors toward nurse managers, such as low narcissism, could be assigned adequate weight [24]. Leadership training and development programs for executive leaders and nurse managers’ direct superiors could also be an approach [22]. These programs should encourage and enable senior leaders and direct supervisors of nurse managers to engage in empowering leadership practices such as promoting innovation, sharing a common purpose, clarifying values, providing opportunities to contribute to strategizing, seeking input on organizational issues, and promoting nurse managers’ self-determination [52]. It could also be valuable to use digital solutions meaningfully to transform the way senior executives and direct supervisors interact with nurse managers into more empowering and less hierarchical, including faster and more direct communication and eye-to-eye collaboration [53].

The study results also suggest that it is important to provide nurse managers with adequate formal rewards. Elements of an engaging financial reward package typically include an appropriate base salary and supplements based on competencies, experiences, and responsibilities, a range of benefits, and performance- and innovation-based pay components [54]. Furthermore, engaging nonfinancial rewards should be provided, including needs-oriented worktime arrangements, educational opportunities to help nurse managers develop management and leadership skills, structured career planning, and mentoring [54]. In designing the reward system, senior management and HR should work closely with nurse managers to consider the specific rewarding needs of nurse managers and develop appropriate and sustainable ways to provide formal rewards.

Finally, it should be considered what opportunities may exist to mitigate the work–life interferences of nurse managers. Recommendations include a restructuring of management structures and roles. One potential option in this regard could be to establish co-management or job-sharing models, where, for example, co-managers can relieve one another on days off or divide responsibilities in such a way that negative effects from role overload are reduced [15,55]. Fostering a family-friendly workplace is another strategy [49] which could include offering worksite kindergartens or other organizational support for childcare. Consideration could also be given to the possibilities of creating flexible working arrangements in terms of time and place and optimizing schedule reliability [56], as this would enable nurse managers to better balance their private and professional lives. Further opportunities to mitigate work–life interferences could arise from hybrid leadership models that combine face-to-face leadership with remote leadership supported by digital technologies [57,58].

### 5.3. Limitations

Our study is one of the few quantitative studies to date that has investigated factors that determine nurse managers’ work engagement. This topic is highly relevant considering the role played by nurse managers in providing healthcare services. However, this study has certain limitations. The cross-sectional design of the study can only show whether there is an association between variables, not a causal relationship. Further, convenience sampling was used to obtain the sample which is inferior to probability sampling in its representativeness of the target population, limiting the external validity of this work. Though efforts were made to include nurse managers of diverse backgrounds, there is a chance that this sampling method may have introduced bias. E.g., nurse managers working in hospitals were likely overrepresented in the sample, which limits the generalizability of our findings. Because the study was conducted in Germany, and given the global importance of the topic, future research could analyze the proposed relationships in other settings to further generalize the current findings. Since the study was conducted online, technology-savvy nurse managers were better reached. There is also the possibility of bias in favor of participation by particularly engaged nurse managers. Further, participation may be influenced by workload. Information biases cannot be ruled out. It is conceivable that nurse managers with high work engagement were more likely to recall abundant resources, whereas participants with lower engagement may have thought more thoroughly about job demands. Multiple answers cannot be ruled out, as no IP addresses were stored and no access codes were used.

## Figures and Tables

**Table 1:** Characteristics of the sample.

	%	n
**Gender**		
Female	70.3	287
Male	29.7	121
**Age (years)**		
≤30	11.3	46
31–40	28.7	117
41–55	42.6	174
≥56	17.4	71
**Type of organization**		
Hospital	88.0	359
Geriatric care facility	12.0	49
**Nursing management position**		
Lower management	70.8	289
Middle management	29.2	119
**Qualification for management position**		
Completed studies or currently studying	30.2	123
Completed further education or currently in further education	57.1	233
No management-related further qualification	12.7	52
**Span of supervision (# of employees)**		
≤25	42.4	173
26–50	28.4	116
51–150	20.1	82
≥151	9.1	37
**Experience in nursing profession (years)**		
≤10	13.0	53
11–20	33.1	135
≥ 21	53.9	220
**Experience in management position (years)**		
≤5	43.6	178
6–14	30.2	123
≥15	26.2	107
**Direct nursing care**		
Yes	66.9	273
No	33.1	135

Note: N = 408.

**Table 2:** Means, standard deviations, Pearson’s correlations, and Cronbach’s alphas of study variables.

Variables	M	SD	1	2	3	4	5	6	7	8	9	10	11	12	13	14	15	16	17	18	19	20	21	22	23	24	25	26	27	28	29	30
1. Lack of formal rewards	2.32	0.51	***0.76***																													
2. Work overload	3.48	0.52	0.249	***0.79***																												
3. Work-life interferences	2.88	0.86	0.376	0.498	***0.89***																											
4. Emotional demands	4.09	0.68	0.160	0.328	0.312	***0.71***																										
5. Role conflicts	3.14	0.70	0.374	0.401	0.450	0.309	***0.77***																									
6. Autonomy	3.65	0.68	−0.359	−0.213	−0.301	−0.076	−0.303	***0.80***																								
7. Professional resources	2.91	1.09	−0.513	−0.379	−0.369	−0.165	−0.418	0.261	***0.79***																							
8. Empowering leadership	4.34	0.84	−0.664	−0.269	−0.384	−0.121	−0.448	0.396	0.574	***0.90***																						
9. Relationships w/nursing staff	5.10	0.78	−0.162	0.041	−0.142	0.088	−0.148	0.154	0.109	0.172	***0.88***																					
10. Relationships w/medical staff	4.47	1.19	−0.130	0.054	−0.042	0.092	−0.189	0.089	0.053	0.129	0.331	***0.82***																				
11. Supervisor support	4.39	1.26	−0.574	−0.206	−0.277	−0.074	−0.347	0.294	0.397	0.692	0.151	0.140	***0.94***																			
12. Relations w/colleagues	4.11	0.57	−0.244	−0.092	−0.234	0.016	−0.133	0.243	0.151	0.204	0.361	0.120	0.171	***0.80***																		
13. Information & communication	3.31	0.84	−0.526	−0.243	−0.322	−0.101	−0.452	0.302	0.430	0.658	0.138	0.166	0.593	0.247	***0.86***																	
14. Gender ^a^	0.30	0.46	−0.046	−0.120	−0.101	−0.066	0.012	0.062	0.107	0.094	−0.100	−0.049	0.037	−0.003	0.124	*--*																
15. Age ≤30 ^a^	0.11	0.32	−0.123	0.004	−0.045	−0.080	−0.047	−0.009	0.059	0.040	0.023	<0.001	0.035	0.104	0.127	−0.011	*--*															
16. Age 41–55 ^a^	0.43	0.50	0.078	−0.041	−0.096	−0.018	−0.033	−0.006	−0.013	−0.041	−0.002	0.016	0.017	−0.038	0.008	0.015	−0.307	*--*														
17. Age ≥56 ^a^	0.17	0.38	0.064	−0.001	0.034	0.023	0.013	−0.039	−0.137	0.023	−0.039	0.082	−0.002	−0.060	0.022	0.084	−0.164	−0.396	*--*													
18. Type of organization ^a^	0.12	0.33	−0.077	−0.027	0.020	0.037	−0.047	0.034	0.083	0.042	−0.088	−0.015	−0.015	−0.104	0.047	−0.091	0.059	−0.029	−0.150	*--*												
19. Management position ^a^	0.29	0.46	−0.120	−0.124	−0.002	−0.034	0.027	0.128	−0.002	0.128	−0.169	−0.183	0.013	−0.055	0.013	0.044	−0.024	0.090	−0.010	0.294	*--*											
20. Qualification: studies ^a^	0.30	0.46	−0.116	−0.049	−0.017	−0.040	0.102	0.070	−0.039	0.090	−0.124	−0.268	0.005	0.079	−0.002	0.100	0.188	−0.070	−0.118	0.020	0.354	*--*										
21. Qualification: no further ^a^	0.13	0.33	0.050	0.024	0.061	<0.001	−0.037	−0.164	−0.018	−0.145	0.076	0.035	0.011	−0.009	−0.050	−0.119	0.050	0.027	−0.098	−0.028	−0.197	−0.251	*--*									
22. Employees 26–50 ^a^	0.28	0.45	0.034	0.006	0.007	−0.028	−0.055	0.029	0.015	−0.021	0.035	0.137	−0.036	−0.047	0.032	0.019	0.033	−0.038	0.026	0.018	−0.082	−0.177	−0.045	*--*								
23. Employees 51–150 ^a^	0.20	0.40	−0.115	−0.060	−0.121	−0.064	−0.057	0.134	0.018	0.092	−0.067	−0.109	0.100	−0.035	0.102	0.023	−0.024	0.062	−0.069	0.078	0.311	0.190	−0.082	−0.316	*--*							
24. Employees ≥151 ^a^	0.09	0.29	−0.104	−0.052	0.065	0.055	0.098	0.070	0.066	0.110	−0.167	−0.205	−0.023	−0.005	−0.005	0.169	−0.086	0.038	0.080	−0.090	0.455	0.351	−0.121	−0.199	−0.158	*--*						
25. Nursing profession ≤10^a^	0.13	0.34	−0.105	−0.040	−0.006	−0.070	−0.006	−0.027	−0.007	0.046	−0.073	0.070	−0.047	0.021	0.048	−0.011	0.738	−0.259	−0.177	0.149	−0.023	0.159	0.049	0.047	−0.030	−0.097	*--*					
26. Nursing profession 11-20^a^	0.33	0.47	−0.064	−0.002	0.045	0.080	0.039	0.060	0.130	0.059	0.079	−0.176	0.011	0.054	−0.056	−0.069	−0.119	−0.301	−0.309	0.061	−0.096	0.060	0.012	−0.004	−0.041	−0.023	−0.272	*--*				
27. Management 6–14 ^a^	0.30	0.46	0.068	0.016	−0.043	0.047	−0.010	−0.028	0.043	0.049	0.069	0.015	0.050	0.016	0.008	−0.006	−0.200	0.233	−0.132	−0.013	0.001	−0.047	−0.075	−0.071	−0.010	0.034	−0.222	0.083	*--*			
28. Management ≥15 ^a^	0.26	0.44	0.056	−0.069	−0.013	−0.018	−0.040	0.052	−0.069	0.013	−0.084	0.038	−0.026	−0.024	0.038	0.125	−0.213	0.060	0.535	−0.169	0.120	−0.052	−0.144	0.044	0.021	0.161	−0.230	−0.407	−0.392	*--*		
29. Direct nursing care ^a^	0.33	0.47	−0.126	−0.228	−0.165	−0.170	−0.085	0.154	0.067	0.174	−0.219	−0.194	0.044	−0.067	0.084	0.159	−0.037	0.078	0.076	0.045	0.534	0.367	−0.144	−0.131	0.349	0.431	−0.008	−0.162	−0.019	0.185	*--*	
30. Work engagement	4.67	1.08	−0.409	−0.133	−0.289	−0.010	−0.285	0.281	0.272	0.425	0.240	0.174	0.352	0.237	0.309	−0.096	0.093	0.039	−0.087	0.020	−0.026	−0.046	0.072	0.031	−0.006	0.001	0.058	−0.014	−0.039	−0.029	−0.003	***0.85***

Notes: N = 408; M = Mean; SD = Standard deviation; ^a^ dummy-coded; gender: 1 = male; comparison category age: 31–40 years; type of organization: 1 = geriatric care facility; nursing management position: 1 = middle management; comparison category qualification: further education; comparison category span of supervision: ≤25 employees; comparison category experience in nursing profession: ≥21 years; comparison category experience in management position: ≤5 years; direct nursing care: 1 = no; all |r| > 0.097 are significant at the level of 0.05, all |r| > 0.127 are significant at the level of 0.01; Cronbach’s alphas for multi-item scales are shown in bold and italics in the diagonal in the correlation matrix.

**Table 3:** Results of the regression analysis.

	Work Engagement				
	B	SE(HC3)	β	t	*p*
**Independent variables**					
Lack of formal rewards	−0.322	0.134	−0.151	−2.406	0.017
Work overload	0.014	0.126	0.007	0.114	0.909
Work–life interferences	−0.149	0.070	−0.120	−2.137	0.033
Emotional demands	0.123	0.083	0.077	1.477	0.141
Role conflicts	−0.063	0.090	−0.041	−0.692	0.489
Autonomy	0.139	0.083	0.087	1.678	0.094
Professional resources	−0.044	0.064	−0.045	−0.688	0.492
Empowering leadership	0.353	0.111	0.276	3.176	0.002
Relationships with nursing staff	0.105	0.080	0.075	1.313	0.190
Relationships with medical staff	0.029	0.046	0.032	0.635	0.526
Supervisor support	0.034	0.066	0.040	0.511	0.610
Relations with colleagues	0.136	0.100	0.073	1.370	0.171
Information and communication	−0.070	0.093	−0.055	−0.755	0.450
**Control variables**					
Gender ^a^	−0.261	0.108	−0.111	−2.417	0.016
Age ≤30 ^b^	0.150	0.240	0.044	0.625	0.532
Age 41–55 ^b^	0.075	0.179	0.034	0.417	0.677
Age ≥56 ^b^	-.214	0.230	−0.075	−0.931	0.352
Type of organization ^c^	0.076	0.178	0.023	0.426	0.670
Management position ^d^	−0.155	0.150	−0.066	−1.037	0.301
Qualification: studies ^e^	−0.129	0.133	−0.055	−0.970	0.333
Qualification: no further ^e^	0.282	0.155	0.088	1.817	0.070
Employees 26–50 ^f^	0.102	0.121	0.043	0.847	0.397
Employees 51–150 ^f^	0.001	0.155	0.000	0.006	0.996
Employees ≥151 ^f^	0.300	0.278	0.080	1.082	0.280
Nursing profession ≤10 ^g^	0.028	0.282	0.009	0.099	0.921
Nursing profession 11–20 ^g^	−0.132	0.192	−0.058	−0.688	0.492
Management 6–14 ^h^	−0.123	0.140	−0.052	−0.879	0.380
Management ≥15 ^h^	0.013	0.180	0.005	0.073	0.942
Direct nursing care ^i^	−0.022	0.150	−0.010	−0.146	0.884
**Model Statistics**					
R^2^	0.312				
Adjusted R^2^	0.260				
F	5.924				
*p*	<0.001				

Notes: N = 408; ^a^ dummy-coded, 1 = male; ^b^ dummy-coded, comparison category age 31–40 years; ^c^ dummy-coded, 1 = geriatric care facility; ^d^ dummy-coded, 1 = middle management; ^e^ dummy-coded, comparison category qualification: further education, ^f^ dummy-coded, comparison category span of supervision: ≤25 employees; ^g^ dummy-coded, comparison category experience in nursing profession: ≥21 years; ^h^ dummy-coded, comparison category experience in management position: ≤5 years; ^i^ dummy-coded, direct nursing care: 1 = no.

## Data Availability

The data presented in this study are available on request from the corresponding author.
